# Identification and evaluation of barriers and facilitators to formal help-seeking for premenstrual symptoms in the UK: a mixed methods approach

**DOI:** 10.3389/fgwh.2026.1740226

**Published:** 2026-04-17

**Authors:** E. L. Funnell, N. A. Martin-Key, C. Jones, E. Wise, K. Babbitt, S. Bahn

**Affiliations:** 1 Cambridge Centre for Neuropsychiatric Research (CCNR), Department of Chemical Engineering and Biotechnology, University of Cambridge, Cambridge, United Kingdom; 2 Department of Psychology, University of Cambridge, Cambridge, United Kingdom

**Keywords:** BACE, barriers & facilitative factors, menstrual health, PMDD, PMS, PSST, women's health

## Abstract

**Introduction:**

Premenstrual symptoms are common physical, psychological, and behavioural symptoms that occur before the onset of menstruation. Despite their cyclical nature, these symptoms can be severe and burdensome, potentially interfering with daily life. Although interventions are available, help-seeking remains infrequent, raising questions about the barriers to engaging in formal care. This study employed a mixed-methods approach to examine both barriers and facilitators to help-seeking and assess their relative impact.

**Methods:**

An online survey was developed, incorporating questions about premenstrual symptoms and quantitative and qualitative questions on barriers and facilitators to formal help-seeking. Participants included both previous help-seekers and non-help-seekers to allow for group comparisons. Data were collected from 592 UK-based participants. Quantitative data were analysed descriptively, with group differences assessed using Mann–Whitney U and Chi-square tests, as appropriate. Qualitative responses were analysed using thematic analysis.

**Results:**

42.74% (*n* = 253) had not previously sought formal help for premenstrual symptoms, while 57.26% (*n* = 339) had. Overall, the most common and strongest barrier to help-seeking regarded concerns that healthcare professionals (HCPs) would not take symptoms seriously or would act dismissively. Significant group differences were observed in the barriers endorsed, with non-help-seekers being more likely to think professional care would be ineffective and wanting to solve problems on their own. Previous help-seekers were more likely to endorse previous poor care experiences as a barrier. Qualitative analysis revealed that anticipated HCP knowledge and attitudes, along with lack of awareness and education, were the most frequently reported barriers. Among non-help-seekers, improving education and awareness was commonly cited as a potential facilitator of formal help-seeking.

**Discussion:**

Concerns of being dismissed or not taken seriously by HCPs was the most influential factor in deciding whether to seek help or not. Additionally, lack of awareness and education was identified as a key barrier, including uncertainty about whether symptoms were “severe” enough to justify seeking formal care and doubt regarding the range and efficacy of treatment options. To facilitate help-seeking, efforts should focus on improving education for individuals experiencing premenstrual symptoms and enhancing the quality of care interactions to address concerns about poor care experiences.

## 1 Introduction

Premenstrual symptoms occur in the luteal phase of the menstrual cycle and typically begin to improve with the start of menstrual bleeding. Symptom manifestations are highly variable between individuals, including psychological, physical, and behavioural symptoms. Frequently reported changes include cravings, mood swings, anxiety, and fatigue (1). These symptoms, whilst cyclical, can be a substantial burden on the individual. Pharmacological and psychological interventions are available to ameliorate premenstrual symptoms and disorders, but access to these interventions requires help-seeking, which can be infrequent for these problems (2).

Reported barriers to help-seeking for women's health in general include difficulties in securing a referral or gaining access to specialist health services (3), concerns of stigma (4) and discomfort discussing certain aspects of health and wellbeing with healthcare professionals (HCPs) (3). Perceptions of previous poor care experiences or concerns about dismissal by HCPs may also play a role (5), with evidence demonstrating that such dismissal is commonplace in care interactions for premenstrual symptoms (6). This dismissal may necessitate individuals to engage in repeated long-term self-advocacy to receive a diagnosis and treatment which can be taxing for those already struggling with health problems (3).

Whilst qualitative investigations have identified factors which may delay or discourage help-seeking (7, 8), to our knowledge, there remains a lack of mixed methods approaches combining qualitative and quantitative analyses examining the relative impact of these factors. Further, much of the research available focuses on premenstrual disorders rather than premenstrual symptoms more broadly. Since premenstrual symptoms can be impairing or distressing even in the absence of a formal diagnosis, it is important to understand the broader barriers and facilitators to help-seeking, particularly as support needs may not always align with established diagnostic criteria. Identifying and evaluating existing barriers is crucial to overcome them ensuring that individuals are encouraged to engage with appropriate services. As such, utilising measures that examine barriers to help-seeking and their impact may yield key insights into the factors most frequently and significantly hindering access to care. Such insights may inform the development of targeted interventions or policies that enable individuals experiencing premenstrual symptoms to access effective treatments.

Therefore, the aims of the current study were to identify and evaluate barriers and facilitators to seeking and accessing formal care specifically for premenstrual symptoms in the UK using a mixed-methods approach. Within the current study, premenstrual symptoms refer to a range of experiences, including premenstrual dysphoric disorder (PMDD), premenstrual syndrome (PMS), and other presentations which do not necessarily meet diagnostic caseness.

## 2 Methods

### 2.1 Participants

Participants were recruited via free and paid posts, as well as advertisements on social media between approximately the 10th of January and the 26th of February 2024. Inclusion criteria were: (1) ≥18 years old, (2) having a strong comprehension of English, (3) assigned female at birth (due to the biological basis of premenstrual symptoms), (4) experience premenstrual symptoms (including but not limited to those with a formal diagnosis of PMS or PMDD), (5) not currently pregnant, perimenopausal or post-menopausal, and (6) not diagnosed with any gynaecological conditions. Premenopausal status was established via self-report during the consent process. As part of the inclusion criteria, participants were required to confirm that they were not currently in the menopause or the menopausal transition (perimenopause), as well as not having been diagnosed with a gynaecological condition (e.g., endometriosis, polycystic ovary syndrome). Only participants meeting this criterion were eligible to participate in the study.

### 2.2 Materials and procedures

An online survey was generated and delivered using the survey software Qualtrics XM®. The survey took 10–20 min, and was adaptive so only relevant questions were displayed based on previous answers. The survey was designed in discussion with an experienced consultant psychiatrist (SB).

All participants were asked about sociodemographic characteristics (age, gender identity, and ethnicity) and premenstrual symptom presence and severity as measured by the Premenstrual Symptom Screening Tool [PSST (9)]. The PSST is a retrospective screening tool comprised of 19-items to assess for premenstrual symptoms and associated functional impairment. In order to more closely align the PSST with Diagnostic and Statistical Manual [5th edition, text revision; DSM-5-TR (10)] criteria, we included a more specific timeframe of symptoms in the question. As impaired romantic or intimate relationships and suicidality are both often associated with PMDD (10), this was added as additional item, therefore, increasing the total number of items to 21. All 21-items were scored on a scale of “Not at all”, “Mild”, “Moderate”, and “Severe”. For the functional impairment items, the “Not at all” scale point was modified to “Not at all/not applicable”. A yes/no question was asked to assess whether premenstrual symptoms occurred in consecutive menstural cycles. We note that retrospective reporting may be subject to recall bias, which could potentially inflate symptom ratings.

Barriers to formal help-seeking were evaluated using an adapted version of the Barriers to Accessing Care Evaluation (BACE) scale (11). The BACE includes 30-items scored on a scale from “Not at all” to “A lot”. If the participant reported never having seen a HCP for premenstrual symptoms they were asked: “Have any of these issues stopped you from getting professional care for your premenstrual symptoms?”. If the participant had seen a HCP for their premenstrual symptoms they were asked: “Did any of these issues initially delay or discourage you from getting, or continuing with, professional care for your premenstrual symptoms?” An additional 6 items were added, expanding on existing items of the BACE (e.g., adding a secondary item to measure previous poor care experiences for gynaecological health conditions in addition to the existing item measuring previous poor care experiences for mental health conditions). These new items were based on previously identified difficulties related to formal help-seeking for premenstrual symptoms or premenstrual disorders (7, 8). Free text questions were also delivered to participants to identify other potential barriers not captured by the adapted BACE. Furthermore, participants who had not previously sought formal help for premenstrual symptoms were asked an additional open text question as to whether there were any factors which would encourage or motivate them to seek formal help.

### 2.3 Data analysis

While the PSST and BACE were modified to align with the specific aims and context of the present study, it is important to note that the adapted versions were used in an exploratory manner and did not undergo independent psychometric validation. Although internal consistency was assessed, reliability estimates alone do not establish construct validity following substantive scale modification. Accordingly, findings derived from these adapted measures should be interpreted with appropriate caution.

The internal consistency of the PSST with additional questions and the adapted BACE were examined with Cronbach's alpha, conducted in SPSS (version 29.0.1.1). Descriptive data analyses (means, standard deviations, frequencies, and percentages) were conducted in Excel version 2206 (Microsoft Office 365), which was also used to create figures.

Group differences in the adapted PSST and BACE items between help-seekers and non-help-seekers were examined in SPSS (version 29.0.1.1). For the adapted BACE, any responses endorsed as “Not applicable” were excluded from analysis, and each item was recoded into new binary variables: “Present vs. Not present” and “Severe vs. Not severe”. Group differences in ordinal data were explored using Mann–Whitney *U*-tests. Group differences in categorical variables were explored using chi-square tests, and effect sizes were calculated as Cramer's V [*φ*c; small ≤ 0.10, medium ≤ 0.30, large ≤ 0.50 (12);]. Group differences in continuous data were explored using *t*-tests.

Qualitative data was analysed through thematic analysis using the Braun and Clarke framework (13). One author (EF) read and re-read the data to familiarise themselves, before writing a codebook for each dataset (barrriers, facilitators) (EF). Codebooks were reviewed by a second author (NMK) to ensure inclusion of all relevant codes against the data. Qualitative data was coded against the codebook under blinded conditions by two authors (CJ/EW). Any qualitative responses that included “No” (or synonyms), “Not applicable” (or synonyms), or were considered ambiguous or non-specific (i.e., not answering the questions) by both coders (CJ/EW) were labelled as not applicable and excluded from the thematic analysis. This procedure was applied systematically to ensure that theme development was based on substantive and interpretable responses. We acknowledge that excluding non-specific responses may influence the scope of themes identified.

During this process, any missing or modified codes identified by EW and CJ were discussed with a third reviewer (EF) and adjusted as needed. After coding, the data was unblinded and discrepancies in code allocation were resolved in collaboration with EF until consensus was reached. After finalizing the codes, two authors (CJ/EW/KB) independently categorized them into themes under blinded conditions. Following unblinding, discrepancies were resolved through discussion with a third author (EF/KB) until consensus was reached (KB only served as a third reviewer when not involved in the initial theme generation). All authors involved in the qualitative analysis have academic backgrounds in psychology. EF and KB are research assistants, NM-K is a postdoctoral researcher, and CJ and EW are student researchers. EF, NM-K, and KB had previous experience conducting reflexive thematic analyses. All researchers recognized that their backgrounds and research experience may have influenced how they interpreted the data. To this end, multiple researchers were involved at all stages of the qualitative analysis (i.e., codebook development, coding, and theme generation) to reduce potential biases. The researchers acknowledge that all findings are shaped by their perspectives.

### 2.4 Ethical approval

This study was approved by the University of Cambridge Psychology Research Ethics Committee (approval number PRE.2023.117). All participants provided informed consent digitally prior to survey commencement.

## 3 Results

Participants who had completed at least 97% of the survey and endorsed premenstrual symptoms in consecutive menstrual cycles (*N* = 592) were included in the analysis. In the current sample, the internal reliability of the adapted PSST and adapted BACE were both high (*α*=0.92 and *α*=0.87, respectively). 42.74% (*n* = 253) of participants had not previously sought formal help specifically for premenstrual symptoms (i.e., non-help-seekers), with the remaining 57.26% (*n* = 339) reporting that they had previously sought formal help specifically for premenstrual symptoms (i.e., help-seekers).

### 3.1 Sociodemographic characteristics and premenstrual symptoms

For a full summary of sociodemographic characteristics and premenstrual symptom presence and severity see Supplementary Tables S1-S3.

The mean age of the sample was 33.91 (SD = 6.18, range=18–51), with the majority identifying as women (97.30%, *n* = 576) and being white (91.89%, *n* = 544). There was a significant age difference between help-seekers and non-help-seekers, with help-seekers having a higher average age (M = 34.66, SD = 5.88) than non-help-seekers (M = 32.91, SD = 6.42; U = 36,189.00, *p* = .001).

In terms of recorded premenstrual symptoms and associated functional impairment, the most frequently endorsed premenstrual symptom was increased anger/irritability (98.14%, *n* = 581). The symptom most frequently rated as severe was fatigue (48.31%, *n* = 286). In terms of associated functional impairment, the most frequently impaired domain was work/studies (94.26%, *n* = 558), with the most severely impaired domain being romantic or intimate relationships (30.41%, *n* = 180).

There were significant differences in the presence of premenstrual symptoms (endorsed at any level) between help-seekers and non-help-seekers, with the former being more likely to report symptoms (see Supplementary Table S2; *χ*^2^s ≤ 17.836, df = 1, ps ≤ .042). The only exceptions were overeating/food cravings and physical symptoms, which did not significantly differ in presence between groups. Additionally, a higher proportion of help-seekers reported functional impairment (see Supplementary Table S3; X^2^s ≤ 34.871, df = 1, ps ≤ .001) aside from impairment in romantic or intimate relationships, where no significant difference was observed.

### 3.2 Quantitative barriers to help-seeking

#### 3.2.1 Most frequently endorsed barriers

See Table 1 for a summary of the top 10 most frequently endorsed barriers across the entire sample. Overall, the most frequently endorsed barrier to accessing formal care was concerns that a HCP would not take premenstrual symptoms seriously or act dismissively (entire sample: 94.09%, *n* = 557; help-seekers: 95.58%, *n* = 324; non-help-seekers: 92.09%, *n* = 233).

**Table 1:** Top ten most frequently endorsed (to any severity, i.e., mild, moderate, severe) barriers to accessing care from the adapted BACE (*N* = 592).

BACE item	*n*	%
Concerns that a healthcare professional would not take my premenstrual symptoms seriously or act dismissively ^a^	557	94.09
Thinking that professional care would probably not help	515	86.99
Concerns that the available treatments for premenstrual symptoms (e.g., combined contraceptive pill, antidepressants, psychological or talking therapy) would not be effective ^a^	493	83.28
Not thinking that my premenstrual symptoms are severe enough to seek professional care ^a^	471	79.56
Concerns about the treatments available (e.g., medication side effects)	463	78.21
Thinking the premenstrual symptoms would get better on their own without any professional care	454	76.69
Being unsure where I can go to get help	450	76.01
Concerns about wasting a healthcare professional's time^a^	444	75
Wanting to solve problems on my own	439	74.16
Preferring to find information and advice online regarding premenstrual symptoms ^a^	370	62.5

a Researcher generated item.

Significant group differences were found in the frequency of endorsed barriers to help-seeking (*X*2s ≥ 8.66, ps ≤ .003, *Φ*cs ≥ .12), with a higher proportion of non-help-seekers endorsing wanting to solve problems on their own, thinking that premenstrual symptoms would get better without professional care, thinking that they did not have a problem, preferring to get help online, not thinking premenstrual symptoms were severe enough to seek professional help, and having concerns about wasting a HCP time.

On the other hand, a higher proportion of help-seekers endorsed being too unwell to ask for help (prior to help-seeking endeavour, having had previous bad experiences with professional care for gynaecological conditions, and having concerns that seeking help may impact their chances of getting a job (*X*2s ≥ 10.62, ps ≤ .001, *Φ*cs ≥ .13).

#### 3.2.2 Barriers most frequently endorsed as severe

See Table 2 for a summary of the top 10 barriers most frequently endorsed as severe across the entire sample.

**Table 2:** Top ten barriers to accessing care most frequently rated as severe from the adapted BACE (*N* = 592).

BACE item	*n*	%
Concerns that a healthcare professional would not take my premenstrual symptoms seriously or act dismissively ^a^	312	52.7
Thinking that professional care would probably not help	199	33.61
Concerns that the available treatments for premenstrual symptoms (e.g., combined contraceptive pill, antidepressants, psychological or talking therapy) would not be effective ^a^	163	27.53
Not thinking that my premenstrual symptoms are severe enough to seek professional care ^a^	154	26.01
Concerns about wasting a healthcare professional's time^a^	148	25
Concerns about the treatments available (e.g., medication side effects)	116	19.59
Having had previous bad experience with professional care for gynaecological or reproductive conditions ^a^	102	17.23
Being unsure where I can go to get help	98	16.55
Having had previous bad experience with professional care for mental health conditions	93	15.71
Wanting to solve problems on my own	92	15.54

a Researcher generated item.

Concerns that a HCP would not take premenstrual symptoms seriously or would act dismissively was also the barrier most frequently rated as severe (entire sample: 52.70%, *n* = 312; help-seekers: 54.28%, *n* = 184; non-help-seekers: 50.59%, *n* = 128).

Differences in the frequency of severely rated barriers between help-seekers and non-help-seekers were examined (Supplementary Table S5). A significantly higher proportion of non-help-seekers rated the following barriers as severe compared to help-seekers: wanting to solve problems on their own, thinking that the premenstrual symptoms would get better on their own, thinking that professional care would not help, thinking that they did not have a problem, thinking that premenstrual symptoms were not severe enough to seek formal care, and not wanting to waste a HCP's time (*X*2s ≥ 3.96, ps ≤ .046, *Φ*cs ≥ .08).

In contrast, a higher proportion of help-seekers rated previous poor care experience for gynaecological conditions and concerns that they may be seen as “crazy” as severe barriers (*X*2s ≥ 4.30, ps ≤ .038, *Φ*cs ≥ .09).

### 3.3 Thematic analysis

#### 3.3.1 Qualitative barriers to help-seeking (help-seekers: *n* = 142; non-help-seekers: *n* = 121)

**Figure 1:**
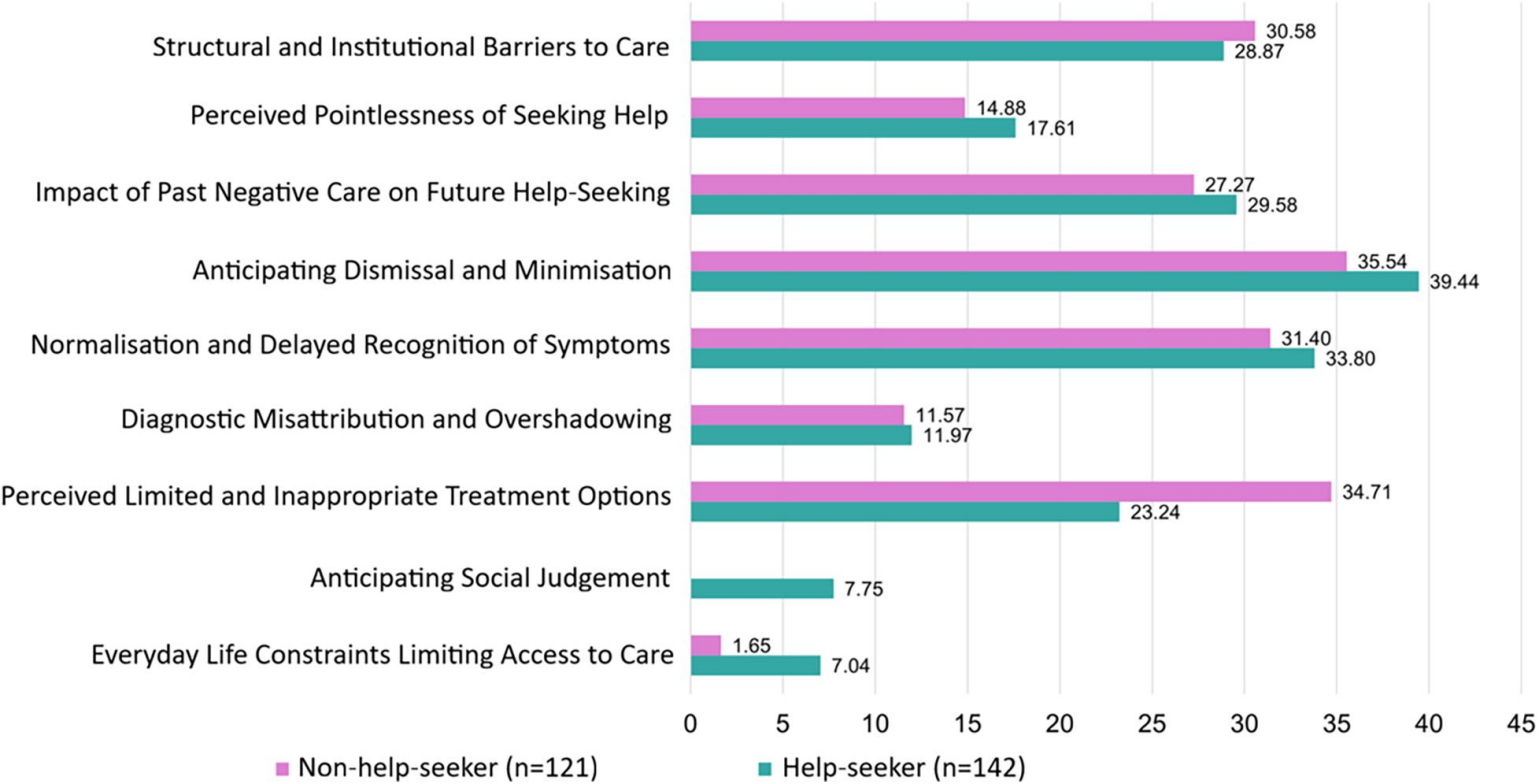
Frequency of themes in the qualitative data regarding barriers to help-seeking in help-seekers (*n* = 142) and non-help-seekers (*n* = 121).

In terms of barriers which were mentioned in the free text data, thematic analysis revealed nine themes comprised of 65 codes (Figure 1).

**Figure 2:**
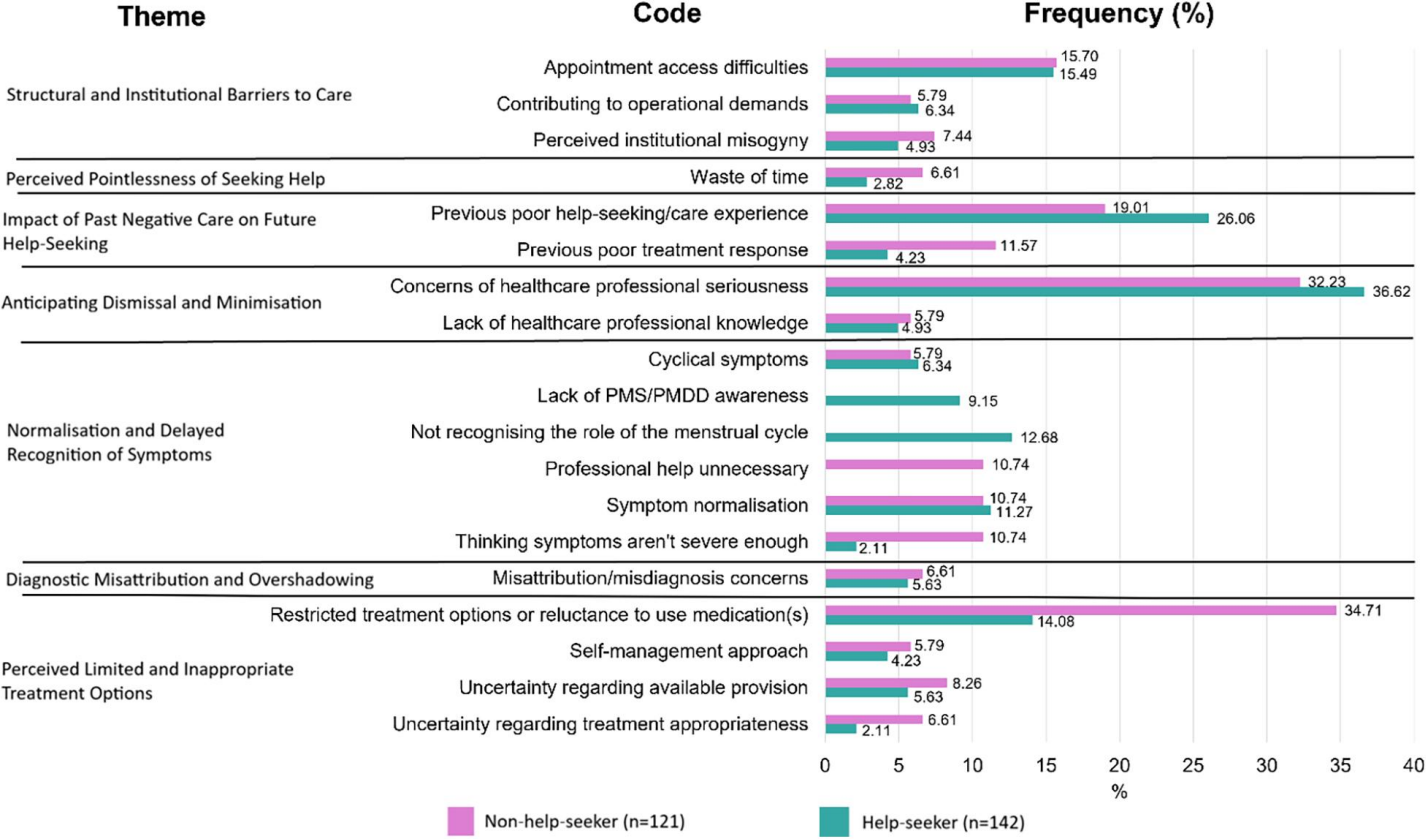
Frequency of codes in each theme identified in the qualitative data regarding barriers to help-seeking in help-seekers (*n* = 142) and non-help-seekers (*n* = 121).

Codes and themes which were present in at least 5% of the free text responses are presented in Figure 2. All codes and frequencies are available in Supplementary Table S6.

##### 3.3.1.1 Anticipating dismissal and minimisation

Participants frequently described concerns that their experiences and symptoms would not be taken seriously by HCPs. For example:

“That I'll just be told its normal and to get on with it” (non-previous help-seeker)

“Knowing that I will more than likely be told that it’s normal, it’s what women have to go through.” (previous help-seeker)

Some reported that these expectation of dismissal caused tem to delay seeking care until their health had significantly deteriorated:*“The dismissal of GP*’*s made me feel shame and therefore deterred me from seeking help until it was usually really severe or I was suicidal.” (previous help-seeker)*

Participants also highlighted that perceived gaps in HCP knowledge could act as a barrier. Several described concerns about the expertise of GPs in premenstrual disorders:

“I would not actively seek support as I do not believe I would see someone with the right expertise say at the GP” (non-help-seeker)

"That GPs don't necessarily know that much about PMDD” (non-help seeker)

Others described needing to initiate treatment discussions themselves, reflecting both a lack of information and a desire for shared decision-making:

“I felt I had to be the one to suggest a solution. I did a lot of research and considered what medication I would feel happy taking before I booked my appointment. The doctor took me seriously but didn’t seem to be aware that antidepressants were an option for PMS.” (previous help-seeker)

##### 3.3.1.2 Diagnostic misattribution and overshadowing

Participants described concerns that their symptoms might be misdiagnosed or attributed to other conditions, particularly when they had a prior mental health history. For example

“Being judged on my mental health history (post partum psychosis) and being told I'm ‘anxious’ or ’stressed’ when I am neither.” (previous help-seeker)

Some participants described experiences of diagnostic overshadowing, where a previous diagnosis appeared to influence HCPs' willingness to consider alternative explanations for their symptoms:

“I sought help for post natal depression and anxiety and I feel like since then GP can be dismissive about health issues putting it down to being anxious.” (non-help-seeker)

##### 3.3.1.3 Perceived limited and inappropriate treatment options

Participants described a perceived lack of available treatment options as a barrier to seeking care, with many expressing reluctance to use medication:

“I feel like, sadly, there is no real solution or support, and that the treatments available are quite limited. Treatments like birth control have been detrimental for me personally, so I feel frustrated and fearful of experimenting with other birth control or with psychiatric medication after the negative experiences I've had with them. Unfortunately, it seems like those are the only two options available through healthcare, so I have given up.” (non-help-seeker)

“I had no desire to start the contraceptive pill” (previous help-seeker)

Non-help-seekers in particular described uncertainty about what treatments were available or appropriate, and a perception that hormonal medication was often the only option:

“I have previously been on the pill and came off it because of my age and the time I had spent on the pill. I don't want to be on it again to managed other symptoms. I don't think it should be the only route of treatment available to women” (non-help-seeker)

“Just the lack of treatment options and the fact that drs don’t necessarily know about PMDD is off putting.” (non-help-seeker)

Some participants also expressed uncertainty about the suitability of available treatments for their symptoms:

“There is also no cure as antidepressants aren’t necessary for three weeks out of four and the pill makes me depressed too.” (non-help-seeker)

Finally, several non-help-seekers described a preference for self-management, choosing to avoid formal healthcare interventions altogether:

“I can manage it fine myself and its not like its going to change the fact that I feel ridiculous sad the week before my period starts. I also don't want to be on medicine or have it in my record” (non-help-seeker)

##### 3.3.1.4 Normalisation and delayed recognition of symptoms

Many participants described assuming their premenstrual symptoms were “normal”, which discouraged them from seeking care:

“Being misinformed about premenstrual symptoms and believing that my issues are ‘normal’” (non-help-seeker)

“I had come to expect the symptoms as part of the menstrual cycle.” (previous help-seeker)

Some participants felt that their symptoms were “*not severe enough”* (previous help-seeker) to warrant professional attention, particularly when the symptoms were cyclical and improved outside of the premenstrual phase:

“It has never seemed relevant or worth the time (for me) to try and get the symptoms checked professionally. I know they are temporary and will pass with the period.” (non-help-seeker)

Cultural and social expectations also influenced help-seeking. Non-help-seekers described feeling that seeking professional help was unnecessary or even inappropriate:

“In my culture it is seen as something normal for all women, that we should be able to deal with it, and that asking for help about menstrual/premenstrual syndromes is an exaggeration and a cry for attention” (non-help-seeker)

For help-seekers, difficulty recognising the connection between the menstrual cycle and mental health symptoms delayed help-seeking. Participants often attributed their experiences to other factors such as work stress or sleep deprivation:

“Maybe there were other reasons I felt the way I did- work stress, sleep deprivation, weight gain.” (Previous help-seeker)

A lack of awareness about premenstrual disorders also contributed to delays:

“It took me a long time to figure out that my depression and tiredness followed the pattern of my cycle because I did not know that PMDD existed.” (Previous help-seeker)

“Becoming aware of the patterns of my behaviour - I just lived like this for years and it was only a chance article about extreme premenstrual symptoms - PMDD that resonated so much with me and my long suffering husband that I then knew it was something the medical profession were aware of.” (Previous help-seeker)

##### 3.3.1.5 Impact of past negative care on future help-seeking

Participants described how negative past experiences with healthcare influenced their future help-seeking. For example, some reported encountering dismissive attitudes or lack of attention to their symptoms:

“Previous experience with nurses. Always offering the pill. Not really caring about the symptoms but more concerned that I could get pregnant.” (previous help-seeker)

“The level of dismissiveness I have encountered since my mid-twenties when I first sought more help for my symptoms for PMDD was huge and is almost singlehandedly the reason why I have not persistently tried to seek more help.” (previous help-seeker)

Both help-seekers and non-help-seekers indicated that these negative care experiences, even if not specific to premenstrual symptoms, shaped their willingness to seek help in the future. Participants also expressed apprehension about accessing care due to previous poor responses to treatments: “*I have no desire to use hormonal treatments as I found these made things worse” (non-help-seeker)*

##### 3.3.1.6 Structural and institutional barriers to care

Participants described a range of structural and institutional barriers that made accessing care difficult. Many highlighted challenges with securing appointments, including long waits, limited booking hours, and reluctance to disclose symptoms to reception staff:

“Difficult to get an appointment; secretary at the doctor’s asks why and I felt like they won't listen to me as to the urgency I feel” (non-help-seeker)

“It is incredibly difficult to get an appointment with any healthcare professional” (previous help-seeker)

Some participants expressed concerns about contributing to operational demands on the healthcare system, feeling that seeking help might take resources from others:

“Feel like I should be in 10/10 pain/psychological distress to feel like not wasting NHS time.” (non-help-seeker)

“During Covid it was not a priority” (previous help-seeker)

Participants also reported a sense that institutional misogyny influenced care, with women's health issues being overlooked or dismissed:

*“General attitudes to menstrual issues and health care system not taking it seriously” (non-help-seeker)*

##### 3.3.1.7 Everyday life constraints limiting access to care

Participants across both groups mentioned everyday life constraits limiting access to care, such as securing time off of work or difficulties related to securing childcare in order to attend appointments:

“Not being able to get through to the Gp practice and then they are not able to offer appointments that did not clash with working hours.” (previous help-seeker)

“Childcare is one of the worst issues aside of the judgement issues. […] I can barely get time to go to the toilet alone, let alone having him quiet for 10 min while I discuss a sensitive subject with someone who may or may not believe me enough to help. Plus, kids shouldn't see their parents being disbelieved or made to cry by other adults.” (non-help-seeker)

##### 3.3.1.8 Perceived pointlessness of seeking help

Participants felt that accessing care would only waste their time, particularly if their concerns were likely to be dismissed or normalised. This perception often led individuals to endure symptoms on their own rather than pursue medical assistance:

“Seeking help is a waste of time and I might as well just white-knuckle through it.” (non-help-seeker)

“It is just wasting my time and making me feel embarrassed becasue he says ‘that’s normal’.” (non-help-seeker)

##### 3.3.1.9 Anticipating social judgement

Some participants described concerns about social judgement affecting their decision to seek help. They reflected on comparisons with others and feelings that their symptoms were something they should manage independently:

“concern I was just ‘making a fuss’ about something other people can handle” (previous help-seeker)

For some, these concerns extended to professional contexts, with participants feeling that seeking support might conflict with their role or expectations at work:

"I'm a psychologist and was very focused on feeling I should be able to manage this anxiety because of my job” (previous help-seeker)

#### 3.3.2 Qualitative facilitators to help-seeking (non-help-seekers: *n* = 156)

To understand if there are any factors that would encourage or motivate formal help-seeking in non-help-seekers, only these were asked about facilitators to formal help-seeking.

**Figure 3:**
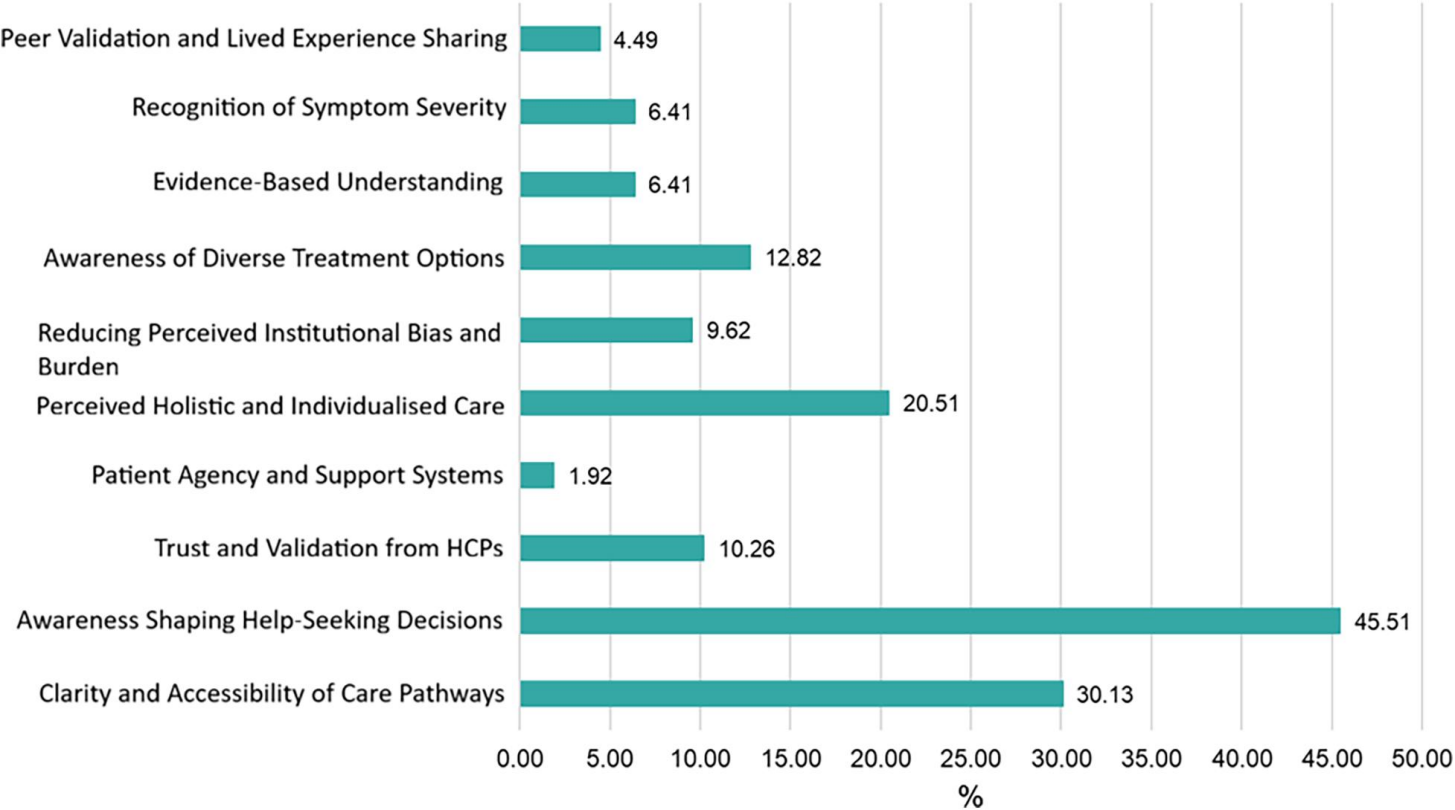
Frequency of themes in the qualitative data regarding facilitators to help-seeking in non-help-seekers (*n* = 156).

**Figure 4:**
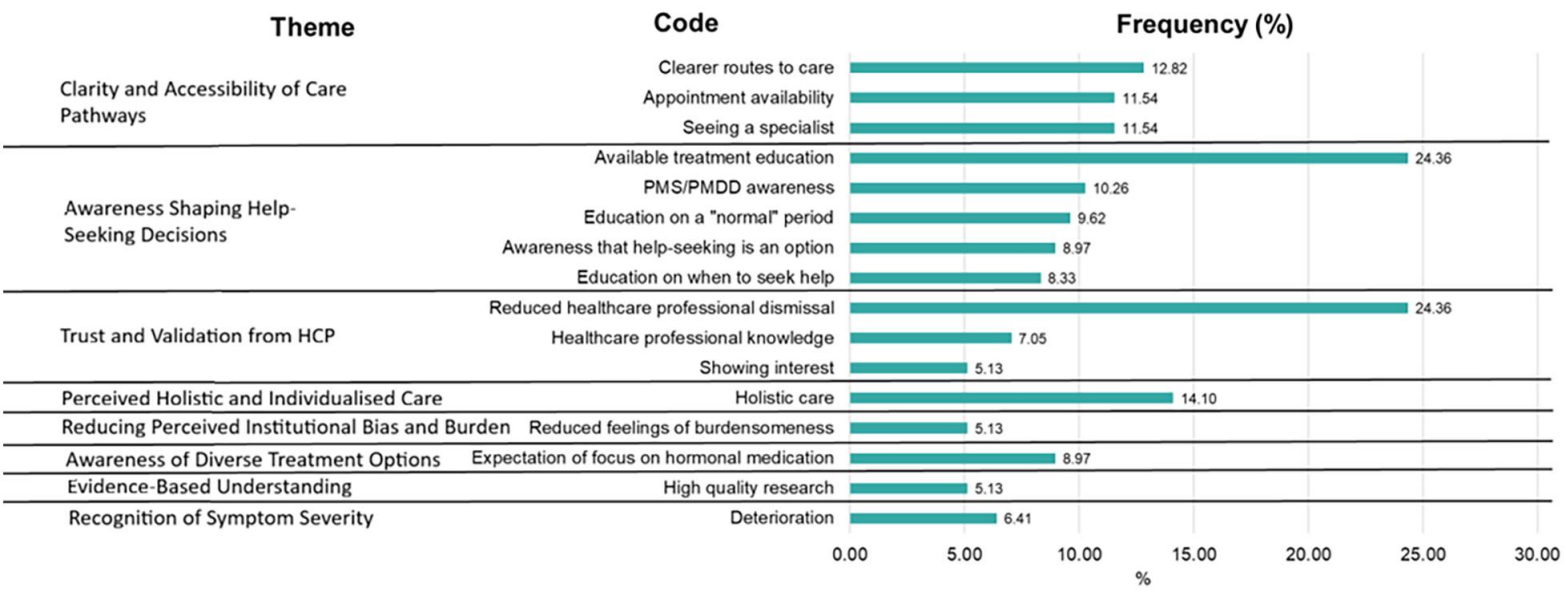
Frequency of codes in each of the themes identified in the qualitative data regarding facilitators to help-seeking in non-help-seekers (*n* = 156).

Thematic analysis revealed ten themes (Figure 3) comprised of 48 codes. Codes and themes which were present in at least 5% of the free text responses are presented in Figure 4. All codes and frequencies are presented in Supplementary Table S7.

##### 3.3.2.1 Awareness shaping help-seeking decisions

Participants described how awareness and knowledge about premenstrual symptoms and available treatments shaped their help-seeking decisions. Many reported uncertainty about when to seek help or what support could be offered:

“I’m not sure when help should be sought, or what help can be offered”

Some participants noted limited awareness of the range of treatment options, often emphasising concerns about over-prescription of hormonal contraceptives:

“Being told what other options are available other than hormonal contraceptives”

“More options of treatment instead of hormonal contraception”

A lack of understanding about more severe premenstrual conditions, such as PMS or PMDD, also influenced decisions to seek care, with some participants feeling their experiences had been normalised and overlooked:

“More awareness of PMDD as a problem - premenstrual symptoms normalised by society so didn't realise what I was experiencing was abnormal for a long time”

Participants suggested that broader awareness efforts could facilitate help-seeking, including media coverage or public health campaigns:

“More media coverage of the premenstrual period and the associated symptoms”.

“A campaign from the NHS” would be a solution to increase “awareness […] of PMS and how awful it makes a lot of women feel.”

Limited knowledge about what constitutes a “normal” menstrual cycle further shaped help-seeking decisions:

“A better understanding of what is ‘normal'”

This lack of knowledge about a “normal” period was reported as a barrier to recognising when to seek help or consider treatment:

“if there was better information out there about PMS and what is normal and what isn't”

Participants also reported that knowing help-seeking was an option encouraged engagement with healthcare:

“Didn’t know that I can treatment for this.”

“Knowing that it is ok to go ‘just’ for that”

“More clarity on what counts as severe enough to warrant visiting a doctor”

##### 3.3.2.2 Awareness of diverse treatment options

Participants stated that removing the expectation of focus on hormonal medication would improve formal help-seeking:

“Knowing that non-hormonal treatment options are available in my area would also be helpful.”

##### 3.3.2.3 Clarity and accessibility of care pathways

Participants also identified a need for “*A clear and manageable pathway”* to care, noting confusion regarding “*what options there are and where”*.

Participants stated that dedicated women’s health clinics would encourage help-seeking, particularly if they did not require a referral:

“if there was a women’s open clinic, with no referral needed.”

“If there was a specific healthcare professional you could go straight to or have drop-in type clinics”

Improving appointment availability and duration was also reported as listed as a facilitator to accessing formal care:

“Ease of getting GP appointments. Appointments being long enough and on time.”

Participants reported that access to specialist care could facilitate help-seeking, but many expressed uncertainty about how to obtain referrals or noted that referrals were typically only offered for severe or persistent symptoms:

“Being able to see a specialist in women’s healthy/fertility/gynaecology rather than wasting my time with a GP”

##### 3.3.2.4 Trust and validation from HCPs

Reducing dismissal by HCPs was a proposed key facilitator to help-seeking:

“Knowing that I would be taken seriously”

“Reassurance from healthcare providers that PMS is a legitimate concern”

This is crucial, as if symptoms are not taken seriously future help-seeking for premenstrual symptoms may be discouraged to the extent that individuals may avoid seeking care altogether:

“No. They are hopeless in this country. Prevention doesn’t exist, they don’t believe the women.”

Participants noted that HCP knowledge is vital, with the need for informed and well-trained clinicians to increase reassurance that they can deliver high-quality care:

“Also assurance that GPs would know how to deal with this.”

“To be reassured that all GP’s have some training and knowledge about this.”

Participants stated that knowing they could easily see a specialist clinician if needed made them more likely to seek help:

“Seeing a specialist who is sympathetic and would listen and not dismiss.”

Participants also emphasised that HCPs simply showing interest was a powerful facilitator of help-seeking:

“If I thought they would show any interest in helping!”

“Knowing there was a GP at the practice with an interest in this area”

##### 3.3.2.5 Perceived holistic and individualised care

The availability of a wider range of treatment options was cited as a factor that increased interest in engaging with formal care, particularly when these options considered individuals' previous responses to treatments:

“If I knew doctors wouldn’t just put me on the pill.”

“Someone who listens and takes on board past experiences—not jumping straight to hormonal contraception or IUD.”

##### 3.3.2.6 Reducing perceived institutional bias and burden

Several participants noting that reduced feelings of burdensomeness such as reducing the perception that they are contributing to strains on healthcare resources would encourage them to seek formal support:

“Better care, stop hearing how ‘busy’ they are and makes you feel unwanted and a burden on the system”

##### 3.3.2.7 Evidence-based understanding

Participants also mentioned that research related to premenstrual symptoms and disorders would incentivise help-seeking:

“For more research to be done about it”

With particular importance placed on research into treatment and management options:

“If there was better evidence for its management”

##### 3.3.2.8 Recognition of symptom severity

For some participants, deterioration of symptoms acted as a key facilitator to help-seeking, prompting them to seek support when their symptoms became particularly severe, started to significantly impact daily functioning, or if they began to experience suicidality:

“Only if I felt really really terrible/suicidal”

“If my symptoms got very severe”

“If they had a more negative impact on my life.”

## 4 Discussion

The current study used a mixed-methods approach to explore barriers and facilitators to seeking formal care for premenstrual symptoms, highlighting the complex interplay of individual, interpersonal, and systemic factors. Across both quantitative and qualitative data, the most consistent concern was the expectation that healthcare providers (HCPs) might be dismissive or fail to take symptoms seriously. Such dismissiveness is well-documented in women's health (3, 14), occurring at multiple stages of care including assessment, diagnosis, and treatment (3). Addressing these perceptions is crucial, as patient experiences of HCP attentiveness influence willingness to seek help and overall care quality (6).

Our findings suggest that perceived HCP knowledge and communication skills are equally important. Some participants noted gaps in HCP understanding of premenstrual disorders and menstruation (15), while positive experiences of informed, attentive providers facilitated help-seeking. Enhancing HCP training in women's health, patient-centred communication, and active listening may therefore improve care interactions and empower patients to access appropriate support (16).

Beyond HCP interactions, decisions to seek help were shaped by personal perceptions and alternative support avenues. Non-help-seekers often managed symptoms independently or used online resources (2), reflecting beliefs that symptoms could be self-managed or were transient. These attitudes may arise from limited awareness of what constitutes a symptom burden warranting professional care. Even among non-help-seekers, symptoms were frequently present, indicating a need for broader public education about premenstrual health to reduce distress and encourage engagement with formal care.

Conversely, help-seekers tended to report higher symptom and functional impairment, with some indicating they were too unwell to initiate care independently. These findings highlight the value of proactive interventions, such as outreach during non-symptomatic phases of the menstrual cycle and symptom tracking tools, which can help individuals anticipate symptom recurrence, identify patterns, and make timely care decisions (17–19). HCP endorsement of these tools may further enhance engagement and facilitate earlier recognition of symptom deterioration, particularly for individuals whose mental health is impacted by cyclical premenstrual symptoms.

Participants also expressed concerns regarding the availability and effectiveness of treatments, particularly the expectation of being offered only hormonal contraception. Improving public and patient education on the full range of evidence-based interventions can support informed decision-making and patient advocacy. Simultaneously, enhancing HCP knowledge and confidence to offer tailored, holistic care ensures that patients perceive formal interventions as relevant and accessible (20–22). Tailoring treatment to symptom profiles and patient preferences is likely to increase engagement and improve outcomes.

Systemic barriers also influenced help-seeking. Difficulties securing appointments, limited access to specialists, and unclear pathways to care were commonly reported. Participants suggested solutions such as “drop-in” women's health clinics, though current UK women's health hubs do not explicitly include premenstrual symptoms as a core service (23, 24). Incorporating premenstrual symptom management into such services could improve accessibility and reduce waiting times, aligning care provision with patient needs.

### 4.1 Limitations

The current study focused on healthcare visits which were specific for premenstrual symptoms, however it is likely that some individuals may discuss premenstrual symptoms with a HCP in the context of another health concern. Therefore, the current findings may have inadvertently excluded care interactions for premenstrual symptoms which occurred in the course of other care (e.g., for another mental health or gynaecological concern).

The majority of the sample were white and identified as women, limiting the generalizability of the findings to individuals from ethnic minority or gender-diverse backgrounds. Given that individuals from minority groups are likely to encounter additional barriers to accessing care (25, 26), caution should be exercised when applying these results more widely. Furthermore, the sample was predominantly highly educated, which may have resulted in fewer barriers to accessing care compared to individuals with lower levels of education. Additionally, age differed between help-seekers and non-help-seekers and may influence symptom severity, pain perception, and the likelihood of seeking care. Although employment status did not differ between the two groups, it may still impact healthcare access. These factors should be considered when interpreting the findings, and future research should formally evaluate their effects.A further limitation relates to the modification of the PSST and BACE for use in the present study. Although adaptations were conceptually guided and internal consistency was acceptable, the modified versions have not undergone independent psychometric validation. Internal reliability alone is insufficient to establish construct validity following substantive scale modification. Additionally, the adapted measures were not subjected to formal pilot testing prior to full data collection. As a result, item clarity, relevance, and acceptability were not independently evaluated before implementation. Accordingly, findings derived from these adapted measures should be interpreted with caution and would benefit from replication using fully validated instruments.

## 5 Conclusion

This study demonstrates that help-seeking for premenstrual symptoms is shaped by interrelated emotional, cognitive, interpersonal, and systemic factors. Key barriers include anticipated HCP dismissiveness, doubts about treatment effectiveness or availability, and uncertainty about symptom severity. Facilitators include supportive HCP attitudes, symptom awareness, education, and accessible, tailored interventions.

Addressing these barriers requires multi-level strategies: improving HCP education and communication skills, enhancing public awareness of premenstrual disorders, expanding knowledge of available interventions, and implementing proactive and accessible care pathways. Together, these approaches can foster more supportive, validating, and effective healthcare experiences for individuals experiencing premenstrual symptoms.

## Data Availability

The dataset presented in this article is not readily available because it is part of ongoing research. Requests to access the dataset should be directed to Prof Sabine Bahn, sb209@cam.ac.uk.
