# Supplementary material for: Identification and evaluation of barriers and facilitators to formal help-seeking for premenstrual symptoms in the UK: a mixed methods approach
Source: Front Glob Womens Health. 2026 Apr 17;7:1740226. doi: 10.3389/fgwh.2026.1740226 (PMC13132999; doi:10.3389/fgwh.2026.1740226)
Supplement: Supplementary file 1 [file Table1.docx]

*Supplementary Materials
Table 1. Summary of sociodemographic characteristics. Key. * Percentage total may exceed 100 as participants were able to select multiple answer options.*

|  | Overall (N=592) | | | Previous help-seeker (n=339) | | | Non-help-seeker (n=253) | | |  |  |  |  |
| --- | --- | --- | --- | --- | --- | --- | --- | --- | --- | --- | --- | --- | --- |
|  | n | % | Mean (SD) | n | % | Mean (SD) | n | % | Mean (SD) | *X* ^2^ (df) | *U* | P | Φc |
| **Age** |  |  | 33.91 (6.18) |  |  | 34.66 (5.88) |  |  | 32.91 (6.42) |  | 36189.00 | .001 |  |
| **Gender** | | | | | | | | | | | | | |
| Woman | 576 | 97.30 |  | 332 | 97.94 |  | 244 | 96.44 |  | 3.194 |  | .272 | .075 |
| Non-binary | 14 | 2.36 |  | 6 | 1.77 |  | 8 | 3.16 |  |  |  |  |  |
| Other | 1 | 0.17 |  | 0 | 0.00 |  | 1 | 0.40 |  |  |  |  |  |
| Prefer not to answer | 1 | 0.17 |  | 1 | 0.29 |  | 0 | 0.00 |  |  |  |  |  |
| **Ethic group** | | | | | | | | | | | | | |
| White or Caucasian | 544 | 91.89 |  | 311 | 91.74 |  | 233 | 92.09 |  | 3.832 |  | .761 | .084 |
| Asian (Indian, Pakistani, Bangladeshi, Chinese, or any other Asian background) | 18 | 3.04 |  | 8 | 2.36 |  | 10 | 3.95 |  |  |  |  |  |
| Black, Caribbean or African | 4 | 0.68 |  | 2 | 0.59 |  | 2 | 0.79 |  |  |  |  |  |
| Hispanic or Latinx | 6 | 1.01 |  | 4 | 1.18 |  | 2 | 0.79 |  |  |  |  |  |
| Mixed or multiple ethnic groups | 17 | 2.87 |  | 11 | 3.24 |  | 6 | 2.37 |  |  |  |  |  |
| Other ethnic group | 2 | 0.34 |  | 2 | 0.59 |  | 0 | 0.00 |  |  |  |  |  |
| Prefer not to answer | 1 | 0.17 |  | 1 | 0.29 |  | 0 | 0.00 |  |  |  |  |  |
| **Highest educational attainment** | | | | | | | | | | | | | |
| Primary education or below (up to 11 years) | 1 | 0.17 |  | 1 | 0.29 |  | 0 | 0.00 |  | 2.628 |  | .942 | .069 |
| Lower secondary education (up to 16 years) | 16 | 2.70 |  | 10 | 2.95 |  | 6 | 2.37 |  |  |  |  |  |
| Upper secondary education (up to 18 years) | 75 | 12.67 |  | 42 | 12.39 |  | 33 | 13.04 |  |  |  |  |  |
| Undergraduate degree | 241 | 40.71 |  | 134 | 39.53 |  | 107 | 42.29 |  |  |  |  |  |
| Postgraduate degree | 245 | 41.39 |  | 142 | 41.89 |  | 103 | 40.71 |  |  |  |  |  |
| Other | 13 | 2.20 |  | 9 | 2.65 |  | 4 | 1.58 |  |  |  |  |  |
| Prefer not to answer | 1 | 0.17 |  | 1 | 0.29 |  | 0 | 0.00 |  |  |  |  |  |
| **Employment*** | | | | | | | | | | | | | |
| Employed (full-time, part-time, self-employed) | 494 | 83.45 |  | 285 | 84.07 |  | 209 | 82.61 |  | .224 (1) |  | .656 | .019 |
| Unemployed | 28 | 4.73 |  | 16 | 4.72 |  | 12 | 4.74 |  | .000 (1) |  | 1.000 | .001 |
| Student | 74 | 12.50 |  | 33 | 9.73 |  | 41 | 16.21 |  | 5.547 (1) |  | .019 | .097 |
| Homemaker | 31 | 5.24 |  | 23 | 6.78 |  | 8 | 3.16 |  | 3.831 (1) |  | 0.050 | .080 |
| Maternity/paternity leave or taking time off of work to care for a family member | 13 | 2.20 |  | 8 | 2.36 |  | 5 | 1.98 |  | .099 (1) |  | .753 | .013 |
| Voluntary work | 17 | 2.87 |  | 5 | 1.47 |  | 12 | 4.74 |  | 5.548 (1) |  | .019 | .097 |
| Prefer not to answer | 2 | 0.34 |  | 2 | 0.59 |  | 0 | 0.00 |  | N/A |  | N/A | N/A |

*Table 2. Comparison of premenstrual symptoms endorsed at any severity (i.e., rated either “mild”, “moderate”, or “severe”) between those who have and have not sought formal help specifically for premenstrual symptoms*

|  | Seen a HCP (n=339) | | Not seen a HCP (n=253) | |  |  |  |
| --- | --- | --- | --- | --- | --- | --- | --- |
|  | n | % | n | % | *X^2^*(df) | P | Φc |
| Anger/irritability | 336 | 99.12 | 245 | 96.84 | 4.119 (1) | .042 | .083 |
| Anxiety/tension | 332 | 97.94 | 240 | 94.86 | 4.192 (1) | .041 | .084 |
| Tearfulness/increased sensitivity to rejection | 336 | 99.12 | 244 | 96.44 | 5.210 (1) | .022 | .094 |
| Depressed mood/hopelessness | 331 | 97.64 | 234 | 92.49 | 8.828 (1) | .003 | .122 |
| Decreased interested in work activities | 322 | 94.99 | 217 | 85.77 | 15.091 (1) | <.001 | .160 |
| Decreased interest in home activities | 314 | 92.63 | 219 | 86.56 | 5.937 (1) | .015 | .100 |
| Decreased interest in social activities | 325 | 95.87 | 227 | 89.72 | 8.689 (1) | .003 | .121 |
| Difficulty concentrating | 328 | 96.76 | 222 | 87.75 | 17.836 (1) | <.001 | .174 |
| Fatigue/lack of energy | 335 | 98.82 | 243 | 96.05 | 4.824 (1) | .028 | .090 |
| Overeating/food craving | 321 | 94.69 | 238 | 94.07 | .106 (1) | .745 | .013 |
| Insomnia | 250 | 73.75 | 148 | 58.50 | 15.290 (1) | <.001 | .161 |
| Needing more sleep | 315 | 92.92 | 217 | 85.77 | 8.131 (1) | .004 | .117 |
| Feeling overwhelmed or out of control | 331 | 97.64 | 232 | 91.70 | 10.974 (1) | <.001 | .136 |
| Physical symptoms | 332 | 97.94 | 244 | 96.44 | 1.227 (1) | .268 | .046 |
| Feeling like you don't want to be alive anymore or feeling suicidal* | 221 | 65.19 | 139 | 54.94 | 6.388 (1) | .011 | .104 |

*Key. * Items were researcher generated for the current study and presented alongside the PSST.*

*Table 3. Comparison of reported functional impairment associated with premenstrual symptoms endorsed to any severity (i.e., rated either “mild”, “moderate”, or “severe”) between those who have and have not sought formal help specifically for premenstrual symptoms*

|  | Seen a HCP (n=339) | | Not seen a HCP (n=253) | |  |  |  |
| --- | --- | --- | --- | --- | --- | --- | --- |
|  | n | % | n | % | *X^2^*(df) | P | Φc |
| Work or studies | 330 | 97.35 | 228 | 90.12 | 13.976 (1) | <.001 | .154 |
| Relationship with coworkers | 264 | 77.88 | 146 | 57.71 | 27.678 (1) | <.001 | .216 |
| Romantic or intimate relationships* | 308 | 90.86 | 220 | 86.96 | 2.284 (1) | .131 | .062 |
| Family | 306 | 90.27 | 203 | 80.24 | 12.087 (1) | <.001 | .143 |
| Social life | 324 | 95.58 | 203 | 80.24 | 34.871 (1) | <.001 | .243 |
| Home responsibilities | 322 | 94.99 | 222 | 87.75 | 10.187 (1) | .001 | .131 |

*Key. * Items were researcher generated for the current study and presented alongside the PSST.*

*Table 4. Comparison of barriers to accessing care endorsed (i.e., “A little”, “Quite a lot“, “A lot”) by those who have and have not sought formal help specifically for premenstrual symptoms. Please note that in an effort to improve readability, bold text is used to highlight any significant group differences.*

|  | Overall sample (N=592) | | Previous help-seeker (n=339) | | Non-help-seeker (n=253) | |  |  |  |
| --- | --- | --- | --- | --- | --- | --- | --- | --- | --- |
|  | n | % | n | % | n | % | *X*^2^ | P | Φc |
| Being unsure where I can go to get help | 450 | 76.01 | 261 | 76.99 | 189 | 74.70 | .416 (1) | .519 | .027 |
| **Wanting to solve problems on my own** | **439** | **74.16** | **234** | **69.03** | **205** | **81.03** | **10.888 (1)** | **<.001** | **.136** |
| Concerns that I might be seen as weak for having a mental health problem | 361 | 60.98 | 202 | 59.59 | 159 | 62.85 | .647 (1) | .421 | .033 |
| Fear of being put in hospital against my will | 117 | 19.76 | 71 | 20.94 | 46 | 18.18 | .697 (1) | .404 | .034 |
| Problems with transport or travelling to appointments | 115 | 19.43 | 62 | 18.29 | 53 | 20.95 | .655 (1) | .418 | .033 |
| **Thinking the premenstrual symptoms would get better on their own, without any professional care** | **454** | **76.69** | **245** | **72.27** | **209** | **82.61** | **8.660 (1)** | **.003** | **.121** |
| Concerns about what my family might think, say, do or feel | 164 | 27.70 | 96 | 28.32 | 68 | 26.88 | .150 (1) | .698 | .016 |
| Feeling embarrassed or ashamed | 318 | 53.72 | 181 | 53.39 | 137 | 54.15 | .033 (1) | .855 | .008 |
| Preferring to get alternative forms of care (e.g., traditional, alternative or complementary therapies) | 214 | 36.15 | 125 | 36.87 | 89 | 35.18 | .180 (1) | .671 | .017 |
| Not being able to afford the potential financial costs involved | 200 | 33.78 | 116 | 34.22 | 84 | 33.20 | 0.67 (1) | .796 | .011 |
| Concern that I might be seen as "crazy" | 329 | 55.57 | 193 | 56.93 | 136 | 53.75 | .592 (1) | .442 | .032 |
| Thinking that professional care would probably not help | 515 | 86.99 | 287 | 84.66 | 228 | 90.12 | 3.814 (1) | .051 | .080 |
| Professionals from my own ethnic or cultural group not being available | 35 | 5.91 | 23 | 6.78 | 12 | 4.74 | 1.086 (1) | .297 | .043 |
| **Being too unwell to ask for help** | **179** | **30.24** | **132** | **38.94** | **47** | **18.58** | **28.473 (1)** | **<.001** | **.219** |
| Concern that people I know might find out | 103 | 17.40 | 59 | 17.40 | 44 | 17.39 | .000 (1) | .997 | .000 |
| Dislike of talking about my feelings, thoughts or emotions | 348 | 58.78 | 189 | 55.75 | 159 | 62.85 | 3.009 (1) | .083 | .071 |
| Concerns that people might not take me seriously if they found out I was having professional care | 235 | 39.70 | 131 | 38.64 | 104 | 41.11 | .367 (1) | .544 | .025 |
| Concerns about the treatments available (e.g., medication side effects) | 463 | 78.21 | 265 | 78.17 | 198 | 78.26 | .001 (1) | .979 | .001 |
| Not wanting a mental health problem to be on my medical records | 221 | 37.33 | 124 | 36.58 | 97 | 38.34 | .192 (1) | .661 | .018 |
| **Having had previous bad experience with professional care for gynaecological or reproductive conditions*** | **345** | **58.28** | **217** | **64.01** | **128** | **50.59** | **10.729 (1)** | **.001** | **.135** |
| Having had previous bad experience with professional care for mental health conditions | 332 | 56.08 | 196 | 57.82 | 136 | 53.75 | .971 (1) | .325 | .040 |
| Preferring to get help from family or friends | 142 | 23.99 | 72 | 21.24 | 70 | 27.67 | 3.284 (1) | .070 | .074 |
| **Thinking I did not or do not have a problem** | **322** | **54.39** | **148** | **43.66** | **174** | **68.77** | **36.843 (1)** | **<.001** | **.249** |
| Concern about what my friends may think, say or do | 87 | 14.70 | 48 | 14.16 | 39 | 15.42 | .182 (1) | .669 | .018 |
| Having no one who could help me get professional help | 173 | 29.22 | 102 | 30.09 | 71 | 28.06 | .287 (1) | .592 | .022 |
| **Preferring to find information and advice online regarding premenstrual symptoms*** | **370** | **62.50** | **192** | **56.64** | **178** | **70.36** | **11.633 (1)** | **<.001** | **.140** |
| **Not thinking that my premenstrual symptoms are severe enough to seek professional care*** | **471** | **79.56** | **239** | **70.50** | **232** | **91.70** | **40.034 (1)** | **<.001** | **.260** |
| **Concerns about wasting a healthcare professional's time*** | **444** | **75.00** | **235** | **69.32** | **209** | **82.61** | **13.641 (1)** | **<.001** | **.152** |
| Concerns that the available treatments for premenstrual symptoms (e.g., combined contraceptive pill, antidepressants, psychological or talking therapy) would not be effective* | 493 | 83.28 | 286 | 84.37 | 207 | 81.82 | .675 (1) | .411 | .034 |
| Concerns that a healthcare professional would not take my premenstrual symptoms seriously or act dismissively* | 557 | 94.09 | 324 | 95.58 | 233 | 92.09 | 3.155 (1) | .076 | .073 |
| **Concerns that it might harm my chances when applying for jobs*^†^** | **155** | **26.18** | **106** | **32.22** | **49** | **19.84** | **10.617 (1)** | **.001** | **.134** |
| Concern that I might be seen as a bad parent*^†^ | 100 | 16.89 | 66 | 28.45 | 34 | 20.24 | 3.753 (1) | .053 | .080 |
| Concern that my children may be taken into care or that I may lose access or custody without my agreement*^†^ | 54 | 9.12 | 36 | 15.72 | 18 | 10.71 | 2.147 (1) | .143 | .060 |
| Difficulty taking time off work*^†^ | 309 | 52.20 | 180 | 56.25 | 129 | 54.20 | .258 (1) | .611 | .021 |
| Concern about what people at work might think, say or do*^†^ | 194 | 32.77 | 117 | 36.22 | 77 | 31.95 | 1.094 (1) | .296 | .043 |
| Having problems with childcare while I receive professional care*^†^ | 90 | 15.20 | 56 | 25.11 | 34 | 20.61 | 1.066 (1) | .302 | .042 |

*Table 5. Comparison of perceived barriers to accessing care endorsed as severe (i.e., “A lot”) by those who have and have not sought formal help specifically for premenstrual symptoms. Please note that in an effort to improve readability, bold text is used to highlight any significant group differences.*

*Key. * Items were researcher generated for the current study and are presented alongside the BACE. *^†^ Total percentage is calculated with those who selected “Not applicable” to the item being excluded.*

|  | Overall sample (N=592) | | Previous help-seeker (n=339) | | Non-help-seeker (n=253) | |  |  |  |
| --- | --- | --- | --- | --- | --- | --- | --- | --- | --- |
|  | n | % | n | % | n | % | *X*^2^ | P | Φc |
| Being unsure where I can go to get help | 98 | 16.55 | 56 | 16.52 | 42 | 16.60 | .001 (1) | .979 | .001 |
| **Wanting to solve problems on my own** | **92** | **15.54** | **44** | **12.97** | **48** | **18.97** | **3.964 (1)** | **.046** | **.082** |
| Concerns that I might be seen as weak for having a mental health problem | 90 | 15.20 | 56 | 16.52 | 34 | 13.44 | 1.066 (1) | .302 | .042 |
| Fear of being put in hospital against my will | 28 | 4.73 | 20 | 5.90 | 8 | 3.16 | 2.410 (1) | .121 | .064 |
| Problems with transport or travelling to appointments | 14 | 2.36 | 9 | 2.65 | 5 | 1.97 | .289 (1) | .591 | .022 |
| **Thinking the premenstrual symptoms would get better on their own, without any professional care** | **80** | **13.51** | **34** | **10.03** | **46** | **18.18** | **8.238 (1)** | **.004** | **.118** |
| Concerns about what my family might think, say, do or feel | 24 | 4.05 | 13 | 3.83 | 11 | 4.35 | .098 (1) | .754 | .013 |
| Feeling embarrassed or ashamed | 51 | 8.61 | 30 | 8.85 | 21 | 8.30 | .055 (1) | .814 | .010 |
| Preferring to get alternative forms of care (e.g., traditional, alternative or complementary therapies) | 18 | 3.04 | 10 | 2.95 | 8 | 3.16 | .022 (1) | .882 | .006 |
| Not being able to afford the potential financial costs involved | 42 | 7.09 | 22 | 6.49 | 20 | 7.91 | .440 (1) | .507 | .027 |
| **Concern that I might be seen as "crazy"** | **57** | **9.62** | **40** | **11.80** | **17** | **6.72** | **4.297 (1)** | **.038** | **.085** |
| **Thinking that professional care would probably not help** | **199** | **33.61** | **104** | **30.68** | **95** | **37.55** | **11.113 (1)** | **<.001** | **.137** |
| Professionals from my own ethnic or cultural group not being available | 7 | 1.18 | 4 | 1.18 | 3 | 1.19 | .000 (1) | .995 | .000 |
| Being too unwell to ask for help | 13 | 2.20 | 9 | 2.65 | 4 | 1.58 | .778 (1) | .378 | .036 |
| Concern that people I know might find out | 10 | 1.69 | 7 | 2.06 | 3 | 1.19 | .674 (1) | .412 | .034 |
| Dislike of talking about my feelings, thoughts or emotions | 55 | 9.29 | 28 | 8.26 | 27 | 10.67 | 1.000 (1) | .317 | .041 |
| Concerns that people might not take me seriously if they found out I was having professional care | 46 | 7.77 | 24 | 6.02 | 22 | 8.70 | .528 (1) | .467 | .030 |
| Concerns about the treatments available (e.g., medication side effects) | 116 | 19.59 | 66 | 19.47 | 50 | 19.76 | .008 (1) | .929 | .004 |
| Not wanting a mental health problem to be on my medical records | 59 | 9.97 | 32 | 9.44 | 27 | 10.67 | .245 (1) | .620 | .020 |
| **Having had previous bad experience with professional care for gynaecological or reproductive conditions*** | **102** | **17.23** | **73** | **21.53** | **29** | **11.46** | **10.305 (1)** | **.001** | **.132** |
| Having had previous bad experience with professional care for mental health conditions | 93 | 15.71 | 61 | 17.99 | 32 | 12.65 | 3.127 (1) | .077 | .073 |
| Preferring to get help from family or friends | 5 | 0.84 | 1 | 0.29 | 4 | 1.58 | 2.861 (1) | .091 | .070 |
| **Thinking I did not or do not have a problem** | **38** | **6.42** | **13** | **3.83** | **25** | **9.88** | **8.818 (1)** | **.003** | **.122** |
| Concern about what my friends may think, say or do | 11 | 1.86 | 6 | 1.77 | 5 | 1.98 | .034 (1) | .854 | .008 |
| Having no one who could help me get professional help | 22 | 3.72 | 12 | 3.54 | 10 | 3.95 | .069 (1) | .793 | .011 |
| Preferring to find information and advice online regarding premenstrual symptoms* | 30 | 5.07 | 12 | 3.59 | 18 | 7.11 | 3.848 (1) | .050 | .081 |
| **Not thinking that my premenstrual symptoms are severe enough to seek professional care*** | **154** | **26.01** | **62** | **18.29** | **92** | **36.36** | **24.591 (1)** | **<.001** | **.204** |
| **Concerns about wasting a healthcare professional's time*** | **148** | **25.00** | **62** | **18.29** | **86** | **33.99** | **19.053 (1)** | **<.001** | **.179** |
| Concerns that the available treatments for premenstrual symptoms (e.g., combined contraceptive pill, antidepressants, psychological or talking therapy) would not be effective* | 163 | 27.53 | 90 | 26.55 | 73 | 28.85 | .386 (1) | .535 | .026 |
| Concerns that a healthcare professional would not take my premenstrual symptoms seriously or act dismissively* | 312 | 52.70 | 184 | 54.28 | 128 | 50.59 | .789 (1) | .374 | .037 |
| Concerns that it might harm my chances when applying for jobs*^†^ | 43 | 7.46 | 30 | 9.12 | 13 | 5.26 | 2.962 (1) | .085 | .071 |
| Concern that I might be seen as a bad parent*^†^ | 28 | 7.00 | 19 | 8.19 | 9 | 5.71 | 1.348 (1) | .246 | .048 |
| Concern that my children may be taken into care or that I may lose access or custody without my agreement*^†^ | 15 | 3.78 | 12 | 5.24 | 3 | 1.79 | 3.251 (1) | .071 | .074 |
| Difficulty taking time off work*^†^ | 70 | 12.54 | 37 | 11.56 | 33 | 13.66 | .630 (1) | .427 | .033 |
| Concern about what people at work might think, say or do*^†^ | 44 | 7.80 | 30 | 9.29 | 14 | 5.81 | 2.315 (1) | .128 | .063 |
| Having problems with childcare while I receive professional care*^†^ | 32 | 8.25 | 20 | 9.97 | 12 | 7.27 | .379 (1) | .538 | .025 |

*Key. * Items were researcher generated for the current study and presented alongside the BACE. *^†^ Total percentage is calculated with those who selected “Not applicable” to the item being excluded.*

Table 6. Barriers identified from thematic analysis of free text data (n=263)

| **Themes** | **Codes** | **Frequency** | **%** |
| --- | --- | --- | --- |
| Anticipating Dismissal and Minimisation | Concerns of healthcare professional seriousness | 91 | 34.60 |
|  | Lack of healthcare professional knowledge | 14 | 5.32 |
|  | Preference to see specialist | 5 | 1.90 |
| Diagnostic misattribution and overshadowing | Misattribution/misdiagnosis concerns | 8 | 3.04 |
|  | Lack of assessment | 6 | 2.28 |
|  | Focus on weight | 5 | 1.90 |
|  | Proving symptom presence | 3 | 1.14 |
|  | Surface level | 1 | 0.38 |
|  | Focus on physical symptoms | 1 | 0.38 |
| Structural and Institutional Barriers to Care | Appointment access difficulties | 41 | 15.59 |
|  | Contributing to operational demands | 16 | 6.08 |
|  | Perceived institutional misogyny | 12 | 4.56 |
|  | Waiting list concerns | 11 | 4.18 |
|  | Short appointment length | 4 | 1.52 |
|  | Perceived care inconsistency | 2 | 0.76 |
|  | Necessary to seek private care | 2 | 0.76 |
|  | Private care is inaccessible | 1 | 0.38 |
|  | Ethnic group | 1 | 0.38 |
|  | Lack of research | 1 | 0.38 |
| Impact of Past Negative Care on Future Help-Seeking | Previous poor care experience | 37 | 14.07 |
|  | Previous poor treatment response | 20 | 7.60 |
|  | Previous incorrect diagnosis | 1 | 0.38 |
| Perceived Pointlessness of Seeking Help | Waste of time | 12 | 4.56 |
|  | Feelings of shame/guilt | 5 | 1.90 |
|  | Reveal a more serious condition | 4 | 1.52 |
|  | Embarrassment | 3 | 1.14 |
|  | Uncertainty describing or discussing symptoms | 3 | 1.14 |
|  | Self-advocacy apprehension | 3 | 1.14 |
|  | Physical exam concerns/reluctance | 3 | 1.14 |
|  | Lack of motivation | 3 | 1.14 |
|  | Perceived as unable to cope | 2 | 0.76 |
|  | Concerns of attention-seeking or hypochondriac perception | 2 | 0.76 |
|  | Fear of section | 1 | 0.38 |
|  | Required to repeat medical history | 1 | 0.38 |
|  | Hesitancy for openness | 1 | 0.38 |
|  | Patient record | 1 | 0.38 |
|  | Lack of healthcare professional familiarity | 1 | 0.38 |
| Everyday Life Constraints Limiting Access to Care | Childcare | 4 | 1.52 |
|  | Time off work | 4 | 1.52 |
|  | Difficulties in symptom tracking | 2 | 0.76 |
|  | Effort of pre-appointment preparation | 1 | 0.38 |
| Normalisation and Delayed Recognition of Symptoms | Cyclical symptoms | 16 | 6.08 |
|  | Symptom normalisation | 29 | 11.03 |
|  | Symptoms would improve on their own | 3 | 1.14 |
|  | Not recognising the role of the menstrual cycle | 18 | 6.84 |
|  | Thinking symptoms aren't severe enough | 16 | 6.08 |
|  | Interaction of premenstrual symptoms and other conditions | 7 | 2.66 |
|  | Uncertainty of whether symptoms are real | 3 | 1.14 |
|  | Lack of PMS/PMDD awareness | 13 | 4.94 |
|  | Unclear routes to care | 5 | 1.90 |
|  | Professional help unnecessary | 13 | 4.94 |
|  | Unaware professional care was available | 11 | 4.18 |
| Perceived Limited and Inappropriate Treatment Options | Restricted treatment options | 34 | 12.93 |
|  | Reluctance to use medication(s) | 27 | 10.27 |
|  | Uncertainty regarding available provision | 18 | 6.84 |
|  | Self-management approach | 13 | 4.94 |
|  | Uncertainty about treatment appropriateness | 3 | 1.14 |
|  | Side effect concerns | 3 | 1.14 |
|  | Lack of treatment optimisation | 2 | 0.76 |
|  | Reluctance to change medication regime | 1 | 0.38 |
|  | Concerns about hormonal medication pressure | 3 | 1.14 |
|  | Existing treatment plan or support provision | 2 | 0.76 |
| Anticipating Social Judgement | Comparison to others | 5 | 1.90 |
|  | Implications on job/work | 4 | 1.52 |
|  | Non-healthcare professional dismissal | 1 | 0.38 |

Table 7. Facilitators identified from thematic analysis of free text data only from previous non-help-seekers (n=156)

| **Theme** | **Codes** | **Frequency** | **%** |
| --- | --- | --- | --- |
| Clarity and Accessibility of Care Pathways | Clearer routes to care | 20 | 12.82 |
|  | Appointment availability | 18 | 11.54 |
|  | Seeing a specialist | 18 | 11.54 |
|  | Women's health clinics | 7 | 4.49 |
|  | Routine appointments | 4 | 2.56 |
|  | Longer appointments | 2 | 1.28 |
|  | Reduced waiting lists | 1 | 0.64 |
| Awareness Shaping Help-seeking Decisions | Available treatment education | 38 | 24.36 |
|  | PMS/PMDD awareness | 16 | 10.26 |
|  | Education on a "normal" period | 15 | 9.62 |
|  | Awareness that help-seeking is an option | 14 | 8.97 |
|  | Education on when to seek help | 13 | 8.33 |
|  | Online resources | 3 | 1.92 |
|  | Help-seeking expectations education | 2 | 1.28 |
|  | Media coverage | 2 | 1.28 |
|  | Educational materials in healthcare settings | 2 | 1.28 |
|  | Online courses from healthcare professionals | 1 | 0.64 |
|  | Educational materials in women's spaces | 1 | 0.64 |
| Trust and Validation from Healthcare Professionals | Reduced healthcare professional dismissal | 38 | 24.36 |
|  | Healthcare professional knowledge | 11 | 7.05 |
|  | Showing interest | 8 | 5.13 |
|  | Attentive listening | 6 | 3.85 |
|  | Gender-inclusive care | 2 | 1.28 |
|  | Healthcare professional gender | 2 | 1.28 |
|  | Reduced focus on weight | 2 | 1.28 |
|  | Trauma-informed care | 1 | 0.64 |
| Patient Agency and Support Systems | Secondary advocate | 1 | 0.64 |
|  | Peer support | 1 | 0.64 |
|  | Access to childcare | 1 | 0.64 |
| Perceived Holistic and Individualised Care | Holistic care | 22 | 14.10 |
|  | Early intervention | 6 | 3.85 |
|  | Holistic assessment | 5 | 3.21 |
|  | Care guarantee | 2 | 1.28 |
|  | Care continuity | 1 | 0.64 |
| Reducing Perceived Institutional Bias and Burden | Reduced feelings of burdensomeness | 8 | 5.13 |
|  | Decreased stigma | 5 | 3.21 |
|  | Perceived institutional misogyny | 2 | 1.28 |
| Awareness of Diverse Treatment | Healthcare professional providing self-help/lifestyle advice | 1 | 0.64 |
|  | Expectation of focus on hormonal medication | 14 | 8.97 |
|  | Side effects | 5 | 3.21 |
|  | Treatment decision involvement | 7 | 4.49 |
| Evidence-Based Understanding | High-quality research | 8 | 5.13 |
|  | Benefit of being involved in research studies | 2 | 1.28 |
| Recognition of Symptom Severity | Onset of suicidality | 2 | 1.28 |
|  | Deterioration | 10 | 6.41 |
|  | Symptom tracking | 1 | 0.64 |
| Peer Validation and Lived Experience Sharing | Peer recommendation or influence | 3 | 1.92 |
|  | Lived experience narratives | 5 | 3.21 |
